# The Life Cycle Transitions of Temperate Phages: Regulating Factors and Potential Ecological Implications

**DOI:** 10.3390/v14091904

**Published:** 2022-08-28

**Authors:** Menghui Zhang, Tianyou Zhang, Meishun Yu, Yu-Lei Chen, Min Jin

**Affiliations:** 1 School of Advanced Manufacturing, Fuzhou University, Fuzhou 350000, China; 2 State Key Laboratory Breeding Base of Marine Genetic Resource, Third Institute of Oceanography, Ministry of Natural Resources, Xiamen 361000, China; 3 College of Ocean Food and Biological Engineering, Jimei University, Xiamen 361000, China; 4 Southern Marine Science and Engineering Guangdong Laboratory, Zhuhai 519000, China

**Keywords:** temperate phage, lysogeny, life cycle transition, regulating factors, ecological implication, prophage induction

## Abstract

Phages are viruses that infect bacteria. They affect various microbe-mediated processes that drive biogeochemical cycling on a global scale. Their influence depends on whether the infection is lysogenic or lytic. Temperate phages have the potential to execute both infection types and thus frequently switch their infection modes in nature, potentially causing substantial impacts on the host-phage community and relevant biogeochemical cycling. Understanding the regulating factors and outcomes of temperate phage life cycle transition is thus fundamental for evaluating their ecological impacts. This review thus systematically summarizes the effects of various factors affecting temperate phage life cycle decisions in both culturable phage-host systems and natural environments. The review further elucidates the ecological implications of the life cycle transition of temperate phages with an emphasis on phage/host fitness, host-phage dynamics, microbe diversity and evolution, and biogeochemical cycles.

## 1. Introduction

**Figure 1:**
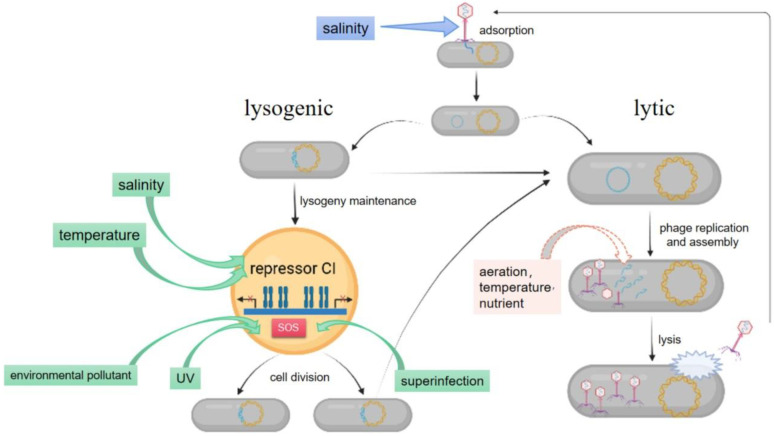
The potential effects of various factors on temperate phage life cycle transition. In the initials of infection, the phage adsorbs to the host cell surface through interacting with host receptors and then injects its genome into the host. Thereafter, the temperate phage enters either the lytic production process or forms a stable coexistence with the host in the lysogenic cycle that is maintained by the repressor protein. Colored arrows indicate factors affecting phage life cycle transition and their potential targets of action. The green solid arrows indicate factors that possibly act on the repressor either directly or indirectly through activation of the host’s SOS response. The pink dashed arrows indicate factors, which are suggested to affect the physiological state of the hosts and thus influence phage life cycle transition, although their effects and underlying mechanisms remain largely unknown. The dark blue solid arrows indicate that salinity may influence the phage life cycle by affecting the phage’s adsorption and genome injection.

Phages are viruses that infect and replicate within bacteria. They are widely distributed in diverse environments (e.g., oceans, soil, and atmosphere) [1], have an extremely high abundance (e.g., 10^5^–10^7^ phage particles/mL in the ocean) [2], possess high morphological diversity (e.g., spherical, icosahedral, filamentous, and tailed) [3], have a large genetic pool with high gene exchange frequency [4,5], complex host-phage interactions, and enormous ecological implications [6,7,8]. Phages are obligate intracellular parasites of hosts and have diverse life cycles (Figure 1). The life cycles include lytic, lysogenic, and pseudolysogenic cycles. In the lytic cycle, the phage starts the production of new viral progeny immediately after infection and releases them by lysing the host. In the lysogenic cycle, the phage genome, known as a prophage, replicates in concert with the host DNA, either integrated into the host’s chromosome or in a free, plasmid-like state, forming a long-term stable coexistence with the host [9,10]. Prophages exit the lysogenic state and enter the lytic cycle, followed by a virion burst under stress conditions [11]. Pseudolysogeny is a non-classical phage life cycle in which phages neither lyse the host nor integrate into the genome to establish a long-term stable relationship [12]. Pseudolysogeny is usually caused by a specific state of the host cell, such as starvation, but turns into the lysogenic or lytic cycles when the condition improves [13]. This review only elucidates the transition of the classical phage life cycles, i.e., the lytic and lysogenic cycles, because pseudolysogeny remains largely unknown. In-depth research on the mechanism of the lysogenic-lytic cycle transition in some phages, such as phage λ, ϕ11, and CTXϕ has been done [1,14]. In phage λ, which is the most studied phage, the phage-encoded CI repressor inhibits the early promoters pL and pR through the formation of CI octamer and DNA loop, thereby maintaining a lysogenic state (Figure 1) [1,15]. The host’s RecA protein is activated to stimulate specific cleavage of the CI repressor upon the induction of the host SOS system, causing the prophage to enter the lysis process [16].

Existing data suggest that lysogeny is widespread, with some studies reporting that more than 90% of the known bacteriophages are temperate phages [17]. Of note, the lysogenic/lytic transition of temperate phage should be of great ecological significance because the life cycle of a phage primary determines the manners, scales, and outcomes of its interaction with the host. The interaction outcomes significantly affect the microbe-mediated ecological processes. For example, the lytic process impacts the microbial community structure and influences the transfer efficiency of nutrients and energy through the marine food web during the “viral shunt” process [6]. In contrast, the ecological impacts of lysogenic processes are more complex and long-term. The impacts affect the evolutionary trajectories of the host, provide immunity against the infection of homologous phages, and facilitate hosts to adapt to hostile environments [3,9,18,19]. The lysogenic/lytic decision of temperate phages is affected by several factors (Figure 1). However, the exact effects of these factors have not been systematically reviewed. A better understanding of the roles of various factors regulating the lysogeny/lytic cycle transition can enhance a comprehensive understanding of the dynamic and ecological implications of temperate phages in continuously changing environments. This review aims to systematically elucidate the effects of various factors on lysogeny/lytic cycle transitions of temperate phages and the potential ecological implications associated with the cycle transitions. 

## 2. Factors Affecting the Temperate Phage Lysogenic-Lytic Cycle Transition

### 2.1. Nutrients

#### 2.1.1. Phosphates

Several studies postulate that lysogeny is more prevalent in oligotrophic offshore waters than in eutrophic coastal waters. This postulate is consistent with the hypothesis that phages prefer lysogenic cycles that coexist mildly within their hosts in the presence of low subsistence resources [20,21]. Inorganic salts of nutrients, such as phosphorus and nitrogen, especially the effects of phosphates, are generally considered important factors affecting the lysogenic-lytic cycle transition of phages [21,22]. On one hand, phosphorus is a growth-limiting nutrient for bacteria; thus, the phosphorus content indirectly affects the life strategy of phages. On the other hand, phages are composed of a high nucleic acid/protein ratio. They thus require sufficient phosphorus for their replication if they enter lytic cycles because of the large-scale burst of viral progeny [23]. 

Numerous field and lab-based studies postulate that changes in phosphorus content significantly affect the abundance of phage particles, which is considered an indicator of phages undergoing lytic production. Wilson et al. infected cultured *Synechococcus* sp. strain WH7803 with isolated temperate S-PM2 phage particles and found that the phage burst size was remarkably reduced, by 80%, under phosphorus-poor conditions compared to phosphorus-rich conditions. Notably, the host lysis rate of the former was only 10% of the latter [24]. In the subsequent study, Wilson et al. observed a large increase in phage particle abundance when exogenous phosphate was artificially added to phosphorus-limited Norwegian fjord mesocosm [25]. Similarly, Tuomi et al. found a significant decrease in bacterial abundance and induction of prophage after adding inorganic phosphates to phosphorus-deficient seawater in Tampa Bay, suggesting that viral activity was stimulated by an increase in phosphate availability [22]. These reports collectively suggest that phages are prone to follow the lysogenic cycle under phosphorus-poor conditions. However, other studies postulated that the effect of phosphorus on phage lysogeny/lysis transition is not fixed and not clear. For example, a study of the Arctic freshwater showed that the bacterial abundance was significantly increased under a combined effect of exogenetic phosphorus and carbon addition, while the phage population did not respond adequately to the same treatment [26]. Only two winter samples with low primary productivity among nine samples in a lysogeny study in Tampa Bay showed successful induction of prophages upon phosphate addition. The authors thus hypothesized that the effect of phosphorus on the phage life cycle is only a part of the “Environmental Impact Complex,” which also includes the impact factors of inorganic nitrogen and chlorophyll *a* [27]. In addition, another lysogeny study in Tampa Bay and the Gulf of Mexico showed that only one of nine observation points had a mild increase of cyanophage particle abundance in response to phosphate addition. In contrast, the remaining eight observation points, including the cultured samples, did not show any special response of the phages to phosphorus addition [22]. Notably, the authors also suggested that mitocycin C, a chemical agent widely used for prophage induction, potentially interfered with the interpretation of the actual effects of phosphorus because it is toxic to bacteria and interacts with the tested inorganic salts [22]. Besides, differences in geographic location, seasons, and methodologies for evaluating lysogeny should also be considered when interpreting results from different studies to assess whether these factors exert a sufficient degree of influence on the results. Genome sequencing has revealed phosphate-stress-related genes in the genomes of some phages. These phosphate-stress-related genes are considered to aid the adaptation of phages to phosphate restriction [28,29]. However, whether they play a special role in the lysogenic/lytic transition remains to be elucidated.

#### 2.1.2. Other Nutrients

Several studies have attempted to explore the possible effects of other nutrients, including nitrogen, carbon, and chlorophyll *a* (an indicator of primary system productivity), on the life cycle decision of phages. Carbon and nitrogen can indirectly affect the lytic production of phages by controlling the host metabolism [30]. The effect of these nutrients on the abundance and productivity of hosts should thus be considered when exploring their effects on the phage life cycle. For example, a study of the microbiological community near the Mediterranean fishery region reported a positive correlation between the abundance of phage-like particles and the content of NO_2_^−^ and heterotrophic prokaryotes. Moreover, the content of total dissolved nitrogen was positively correlated with prokaryotic abundance [31]. Similarly, a mesocosm experiment by Tuomi P et al. at the Gulf of Finland revealed a higher abundance of viral particles when the mesocosm system was supplemented with exogenous inorganic nitrogen, phosphorus, and organic carbon. FVIC (frequency of visibly infected cells), another indicator to estimate the fraction of lytic production, was also highest when the mesocosm system was supplemented with exogenous carbon [32]. 

The chlorophyll *a* content also affects phage lysogenic/lytic cycle transition [27]. For example, studies from the Mediterranean lagoons showed that phages undergoing lytic production exhibited a seasonal pattern in which FVIC was positively correlated with the content of dissolved organic carbon and chlorophyll *a* [33]. Payet et al. also reported that the transition of the phage lysogenic/lytic cycle in the Canadian Arctic Shelf, southern Beaufort Sea, was dependent on system productivity. In the study, lytic infection was significant when system productivity was high, while lysogeny was dominant when system productivity was low [34]. System productivity is tightly linked with prokaryotes’ metabolic activity through its impacts on the dissolved and particulate pool, potentially affecting phage lytic production [35,36]. Several culture studies postulated that phage lytic production is inhibited under starvation conditions and is characterized by a longer latency period and a smaller burst size for phage production, suggesting a strong link between host metabolic status and phage life strategy [37,38]. Generally, nitrogen, carbon, and chlorophyll *a* potentially influence the life cycle decisions of phages though not strongly and directly like phosphorus. 

### 2.2. Salinity 

Salinity and aeration are important factors that affect bacterial growth. They also play a role in the lysogenic/lytic transition of phages by affecting the lysogenic maintenance of phages [39,40,41,42,43]. For example, a study on marine phage ΦHSIC showed that high salinity induced the transition of the phage from the lysogenic to lytic cycle. The absolute abundance of phage particles and their relative abundance to the host increased with salinity (mainly NaCl and MgSO_4_), especially when the salinity exceeded that of the natural environment [39]. Similarly, a study of phage-host interactions in four natural aquatic sites with varying salinity levels in Senegal reported that the abundance of phage particles was positively correlated with salinity, with a decrease in phage diversity under high salinity conditions [40]. A meta-transcriptome analysis of *Microcystis aeruginosa* and its phages also revealed that the expression of lysis-related genes was positively correlated with salinity [42]. In addition, a study of halophage SNJ1 challenged with various NaCl concentrations revealed that high salinity promoted the host-adsorption and proliferation of halophage SNJ1 and decreased the lysogeny proportion [43]. The highlighted studies suggest that salinity is positively correlated with a phage’s preference for lytic production. The lysis-promoting effects of high salinity are attributed to the potential inhibition of the phage repressor activity. Studies postulate that high salinity reduces the affinity of repressors to the target promoters by inhibiting the interactions between the charged amino acid side chain of phage repressor and DNA phosphate [39,44]. 

In contrast, several studies report that phages are prone to enter the lysogenic cycle under high salinity conditions. A study on the seasonal dynamics of phages in Tampa Bay revealed a negative correlation between the abundance of free phage particles and salinity. The peak of phage particle abundance occurred in the rainy season, characterized by low salinity [45]. Similarly, a study on freshwater viruses in the Red River Delta of northern Vietnam found that the number of free phage particles decreased to the minimum at day 7 when salinity increased daily proportionately. The number of phage particles then gradually recovered to an approximate initial state on Day 12 [46]. Notably, phages tend to enter the lysogenic cycle at extremely high salinity (above 250%), according to the aforementioned study from Senegal [40]. The exact reason for this phenomenon is unclear, but it is potentially associated with changes in host morphology, such as an increase in the proportion of atypical square cells, at extreme high salinity, which is positively correlated with lysogeny [41]. The contrasting effects of salinity on the phage life cycle are attributed to the high variability of up to four orders of magnitude in the effect of salt concentration on the adsorption of phages to hosts, depending on the characteristics of each phage [47]. For example, the adsorption capacity of halovirus SNJ1 increases with salinity, phage Hs1 exhibit an opposite trend, while phage SCTP-2 peaks at a certain salinity range [43,48,49]. Phages likely take salinity into account when making life cycle decisions, given that phage adsorption to host is crucial for the successive efficient infection of new hosts in lytic cycle [50].

### 2.3. Aeration

Lysogeny is generally highly prevalent in environments with anaerobic (low aeration) conditions, such as the gut, deep soil, and deep-sea hydrothermal fluids [51,52,53]. Phage-mediated host lysis is usually not a major factor for microbial mortality in these environments [52,54]. Culture-based experiments also suggest a potential link between aeration conditions and phage life cycles. For example, a culture-based study on *Listonella pelagia* and its phage HSIC revealed that the phage’s lytic production was significantly increased at high aeration conditions and vice versa [39]. The authors hypothesized that high aeration improves the host’s metabolic status and cell growth, which favor the lytic cycle. Kudva et al. tested the infection of *E. coli* with three isolated phages in liquid culture and found that culture aeration was crucial for efficient lysis [55]. A study by Sergeant and Yeo on the generation of phage μ2 preparations also revealed that poor aeration led to low phage yields for culture and infection, further highlighting the importance of adequate aeration in enhancing production efficiency [56]. Generally, it is hard to infer a direct link between aeration conditions and a phage life cycle from the existing studies, especially studies conducted in natural environments with complex factors. Nonetheless, it is generally true that adequate aeration conditions favor the transition of phages from the lysogenic to the lytic cycle because it enhances the hosts’ metabolic activities to produce phage progeny [55,57]. 

### 2.4. Ultraviolet Radiation (UV)

UV plays an important role in phage life cycle transition [58,59,60,61,62]. Numerous studies report that UV induces prophage to enter the lytic cycle, although there is a model estimation that UV promotes the decay of free phage particles and reduces phage infectivity, thus making lytic production, not a good strategy for phage survival [58]. As early as 1987, Ackermann et al. discovered that UV effectively induced prophage [59]. Jiang et al. also found that UV at a wavelength of 254 nm efficiently induced prophage in seawater sampled from the Gulf of Mexico and Mamala Bay. In contrast, there was no similar adequate response of phages upon exposure to sunlight [60]. Notably, UV has varying effects on prophage induction, depending on the environment and lysogens. For instance, the proportion of UV-induced lysogens is lower in offshore areas than in coastal areas [60,63]. Moreover, some lysogens do not respond to UV even under laboratory conditions [64]. 

UV damages the host’s DNA, activating the host’s SOS response that induces prophage [65]. The exact mechanism by which the host’s SOS response leads to prophage induction differs in various phage-host systems. For instance, UV-mediated host DNA damage activates RecA, which promotes the automatic cleavage of LexA and CI repressor in phage λ/*Escherichia coli*. The decrease in CI repressor ultimately leads to the termination of lysogeny maintenance and initiates the phage lytic process [1]. LexA is responsible for suppressing the host’s SOS response and is not directly involved in regulating lysogeny maintenance. In contrast, LexA directly represses the expression of phage lysis-related genes by binding to an AT-rich region of PrstA (promoter of *rstA*) in the case of *Vibrio cholerae* and its phage CTX [14]. Notably, the lytic process initiated by the host’s SOS response in phage λ/*E. coli* is irreversible because the synthesis of the CI repressor is blocked when the CI repressor is below the critical amount. However, the induced lytic process in phage CTX/*V. cholerae* is reversible because LexA and RstR (another repressor encoded by phage CTX) can be re-synthesized to re-establish the lysogeny maintenance when the UV treatment is removed [14,66]. Similarly, UV treatment promotes the synthesis of Tum in *Salmonella enterica* Fels-2 phage, which then binds to and antagonizes the activity of coliphage 186 repressor, allowing the prophage to initial lytic productions [67].

### 2.5. Temperature

Temperature is another potentially important factor that affects the life cycle decision of phages. Jiang et al. reported a significant increase in the direct count of free viral particles in 33% of seawater samples from the Gulf of Mexico and Mamala Bay when the temperature was raised from 24 to 30, 37, or 42 °C [60]. A study of a novel group of phages infecting a soil-borne pathogen, *Burkholderia pseudomallei*, revealed that the phages switched their lifestyle according to temperature. The phages predominantly underwent a lytic cycle at a higher temperature (37 °C) but remained temperate at a lower temperature (25 °C) [68]. Similar phenomena have been observed in phages infecting *Lactococcus lactis* [69] and cyanobacteria [70]. Interestingly, several studies suggest that the phage life-cycle strategy exhibit seasonal variation patterns. For example, a survey of the microbial communities in the coastal waters of northeastern Taiwan showed that the phage/host dynamics had significant seasonal characteristics. The total viral abundance and virus-to-bacteria ratio (VBR) were significantly higher in summer than in winter [71]. In another study on the coastal central Red Sea, the proportion of lysogeny was highest in winter, although there was no significant relationship between phage abundance and water temperature [72]. Similarly, seasonal dynamic studies in the Antarctic Salt Lake [73] and Tampa Bay [27] also found higher proportions of lysogeny in winter and spring than in summer. Of note, seasonal variation involves changes in many factors, including temperature, salinity, and primary productivity, suggesting that complex factors may regulate seasonal variation in the phage life cycle. Seasonal dynamic studies thus only imply a potential link between temperature and phage life strategy, as opposed to the factor-restricted-controlled culture studies. 

Denaturation of the phage repressor through the alteration of its structure, as observed in phage λ, is a potential explanation for the effects of high temperature (above 40 °C) on the phage life cycle [74]. Besides, the temperature may influence the phage life cycle strategy by affecting host metabolism and growth through the regulation of enzyme kinetics, molecular diffusion, and membrane transport [26]. Weinbauer et al. revealed that high temperature reduces phages’ infectivity [54] by distorting the structural conformation and elasticity of phage lipid membranes or capsid proteins [47,75], thus may in turn affect phages’ life cycle strategy.

### 2.6. Heavy Metals

Several studies suggest that copper ions in the marine environment significantly induce prophages. In contrast, zinc does not induce a similar effect [76,77,78]. Similarly, a study on freshwater phycophage AS-1 found that copper-induced prophages, but the induction effect decreased over time [78]. The authors attributed the attenuation of copper’s effects either to the stabilization of the phage-host interaction after initial stress or the interruption of phage lytic productions because of the toxic effects of copper on the host [78]. Another study in which *Pseudomonas aeruginosa* PAO1 was exposed to copper oxide nanoparticles revealed that copper oxide induced prophages and affected the transcription pattern of the host. Copper oxide inhibited transcription of most denitrification genes and upregulated metal resistance genes [79]. The highlighted studies provide strong evidence that copper induces prophages.

Copper is toxic to bacteria and extracellular phages [80,81]. Copper has good antibacterial properties despite its mechanism not being fully clear [80]. Several studies postulate that copper also inactivates extracellular phages [82,83,84]. Generally, copper has more influence on RNA phages, such as MS2, and ssDNA phages, such as S13, than dsDNA phages, including phages T1 and T4. Moreover, phages with a lipid envelope are more sensitive to copper, exhibiting more toxicity toward phages in liquid medium [81,82,83]. The existing studies do not discuss the potential role of copper sterilizing effects in phage life cycles. However, it is reasonable to hypothesize that phages are prone to coexist with hosts in a lysogenic manner to avoid the toxicity of copper against free phages.

Besides copper, cadmium significantly induces prophage in *Nitrosospira multiformis* 25196 in a concentration-dependent manner. Low cadmium concentrations cause bacteria mortality through prophage induction, while high cadmium concentrations cause direct cell death through binding to cellular proteins [84]. A recent metagenomic study on the dynamics of phages and hosts in soils collected in Zhangye and Luzhou City of China containing varying degrees of chromium contamination revealed that phages are more likely to be temperate with the increase of heavy metal contamination [85]. Notably, phage-encoded metal resistance genes responsible for microbial heavy metal detoxification were also up-regulated in soil samples with high chromium contamination, besides up-regulation of the lysogenic genes, such as integrase genes [85].

### 2.7. Environmental Pollutants

The effects of environmental pollutants on the growth and survival strategies of microorganisms, including phage life cycles, have been extensively explored [60,86,87,88,89]. For example, Jiang and Paul successfully induced prophages in bacteria isolated from estuarine, coastal, and oligotrophic offshores in the Gulf of Mexico and Mamala Bay using common aromatic and aliphatic hydrocarbons pollutants, including phenanthrene, naphthalene, and pyrene. Among them, the induction efficiency of polycyclic aromatic hydrocarbons reached 73% [60]. Similarly, Cochran et al. used pollutants, such as polychlorinated biphenyls (PCBs), which are a kind of chlorinated aromatic compounds, and pesticide mixtures, to induce a natural bacterial community in the Gulf of Mexico. The pollutants promoted the induction of prophages, with the induction efficiency of PCBs reaching as high as 75% [86]. In the same line, Yoshida et al. found that exposure to relatively low concentrations of heavy oil (10 μg/mL) induced viral lytic production in the surface seawater samples collected from Matsuyama Port, Japan. In contrast, high concentrations of heavy oil (1 mg/mL) reduced both bacterial and viral populations mainly because of high cytotoxicity [90]. The tested inductive agents in the highlighted studies were mostly pollutants produced from industries, agricultural activities, and transportation. Most of them exhibited induction efficiency significantly higher than mitomycin C. A study by Danovaro et al. exploring the effects of living pollutants, sunscreen and solar oil, on the marine microbial community in Portonovo (Ancona, northern Adriatic Sea) revealed that they both induced viral lytic production and led to VBR increase [88]. Remarkably, the induction effects seemed to be long-term (up to 3 months). Danovaro et al. further found that the sunscreen significantly induced the prophages of zooxanthellae, whose subsequent lysis led to coral bleaching [91].

Some pollutants, such as polycyclic aromatic hydrocarbons, are known carcinogens and mutagens that can trigger bacterial SOS responses. Prophage induction by pollutants is thus potentially caused by the activation of the SOS response system in host cells, similar to the action mode of UV [92]. Interestingly, prophage induction by pollutants decreases from eutrophication in the estuarine environment to oligotrophication in offshore environments. This phenomenon is attributed to the synergistic effect of other pollutants in the eutrophic estuarine environment [87]. Alternatively, the actively growing bacteria in the eutrophic environments potentially possess more active DNA replication and repair machinery to trigger prophage induction [86]. Pollutants generally seem to induce prophages. However, lysogeny is favored to establish long-term reciprocal relationships between phage and host in environments where pollutants are in extreme concentrations or long-term pollution persist. For example, a study on long-term arsenic-contaminated soil in Shimen and Xianghualing, China, revealed a widespread presence of lysogenic phages carrying arsenic biotransformation genes [93]. Lysogeny was beneficial for both the phage and the host in this scenario. Lysogeny helped maintain host resources for phages, while the presence of prophage AMGs endowed the host capability to degrade the pollutant.

### 2.8. Superinfection

Lysogens formed by infection with one temperate phage are often immune to superinfection with phages of the same genotype or homologs, conferring corresponding phenotypic changes and longer survival benefits to the hosts [94]. Most mechanisms by which phages develop superinfection immunity involve changes in the host membrane components that specifically block second infection steps. Firstly, they prevent the adsorption of other phages to host cells. For example, the products of the prophage *cor* genes of coliphages Φ80 and N15 block the adsorption of superinfecting phages T1, Φ80, and N15 to the host cell surface [95]. Secondly, they prevent the injection of superinfecting phage DNA. For example, *Salmonella Typhimurium* prophage P22 does not affect the adsorption of superinfected phage P22 through the cell membrane, but the SieA it produces prevents the DNA of P22 from entering the host cell [96]. However, lysogens do not prevent the second infection by different types of phages. Numerous studies report that a host can be infected by multiple heterologous temperate phages [97]. Importantly, secondary infections easily lead to host lysis. Espeland et al. identified the poly-lysogeny of EI Tor-type *V. cholerae* and showed the emergence of efficient prophage induction after re-infection with another temperate phage FP15 [98]. In another study, Basso et al. used two prophages (Φ-A and Φ-D) to infect themselves and each other’s lysogen of the same host. Both lysogens were resistant to the same phage but were lytically infected by the other phage that induced the existing prophage [99]. The cause of prophage induction through secondary infection is not explicit. However, it is suggested that infection from other phages may trigger prophage induction by eliciting an SOS response in the host [100].

The number of phages that infect a specific host cell, referred to as multiplicity of infection (MOI), has long been thought to influence the fate of host cells despite its exact mechanism remaining largely unclear [101]. High MOI favors lysogeny while low MOI favors lysis in most cases, but other factors may also affect the outcome [20]. Interestingly, Erez et al. reported a small-molecule communication system of phages in which a phage produces a short peptide in its early lytic infection [102]. The phage then switches to lysogeny during subsequent infections when the concentration of this signaling molecule reaches a certain threshold. Notably, Erez et al. showed that only half of the cells were lysogenized even at the maximum concentration of the signal peptide, suggesting it was a stochastic event despite an increased probability of lysogeny. In addition, Zeng et al. suggested that each phage that infects a host cell either makes a lysogenic or lytic decision. The cell eventually follows the lysogenic cycle only when all phage particles support lysogeny [103]. The decision-making for determining the outcome of superinfection is complex and elaborately regulated and thus merits further investigation of the underlying mechanism. 

### 2.9. Host Density

Currently, there are two primary theories, namely Kill-the-Winner (KtW) and Piggyback-the-Winner (PtW), put forward to elucidate the relationship between phage life cycle and host density [104]. In the KtW theory, the phage preferentially kills the high-abundance dominant host population, thereby improving the availability of resources and the diversity of the microbial community [105]. KtW is a long-standing theory supported by many studies regarding the natural microbial community [72,73,106] and prophage induction assays [11,33,107,108]. In contrast, the PtW theory proposes that an increase in host density leads to a decrease in VBR, which results in the persistence of dominant populations, thereby reducing microbial diversity [51]. PtW is a young theory that numerous studies have challenged since it was proposed in 2017 [109]. Nonetheless, it provides novel insights into the dynamic of phage/host interaction and is supported by a growing number of studies. For example, a study on *Vibrio anguillarum* and its prophage H20 showed that high cell densities favor lysogeny [110]. Similarly, Lara et al. identified a high proportion of lysogeny in surface seawater with a high prokaryotic abundance compared to deep ocean waters [111]. Despite the ongoing debate between PtW and KtW, there is a perception that KtW and PtW are not mutually exclusive but work together [103,112].

Several studies postulate the existence of a host density-dependent quorum sensing (QS) molecular communication between phages and between phages and hosts, which may determine lysis–lysogeny decisions [102,113]. The signaling molecules of the QS system are usually products of the host or phage, such as short peptides, acyl-homserine lactones (AHLs), and the like. Notably, the concentration of released signaling molecules depends on the host density [114]. For example, Liang et al. found that QS based on the host-phage interaction triggered the induction of prophages in soil bacteria collected from an agricultural field in East Tennessee of USA through experiments that involved the addition of exogenous AHLs [114]. The QS system of *V. cholerae* is composed of a phage-derived VqmA and a host-derived DPO (3,5-dimethylpyrazin-2-ol). A combination of VqmA and DPO activates the expression of qtip, which sequesters the phage CI repressor, leading to prophage induction [113]. In contrast, another study showed that QS leads to increased lysogeny at high densities of *Vibrio anguillarum*, inhibition of cellular biofilm formation, and increased proteolytic activity [109]. The host-density-dependent QS system reflects the close relationship between the host density and phage life cycles and may thus help to better understand different phage-host dynamics, including KtW and PtW. 

## 3. Potential Ecological Implications of Lysogenic/Lytic Transition

### 3.1. The Transition from Lytic to Lysogenic Cycles

**Figure 2:**
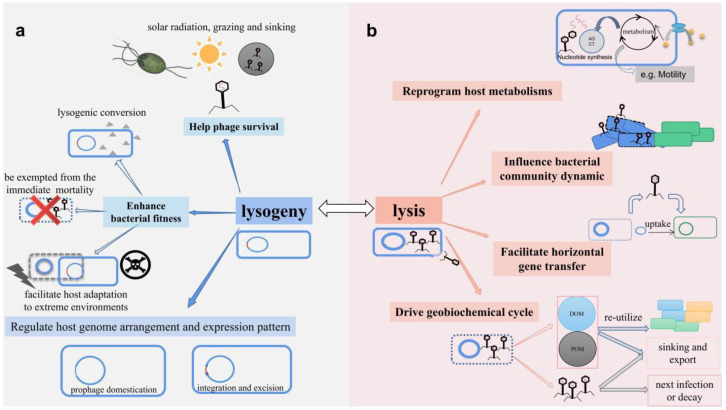
Potential ecological implications of lysogenic/lytic transition. (**a**) Potential ecological implications of lytic to lysogenic cycle transition. The transition protects free phages from various stresses such as solar radiation, grazing, and sinking by attaching to marine aggregates. Lysogeny also enhances bacterial fitness through lysogeny conversion, which averts immediate mortality and facilitates host adaptation to extreme environments. Moreover, lysogeny regulates the host’s genome arrangement and expression pattern through integration and excision of prophages. The red cross symbol represents that hosts are exempted from immediate phage-mediated lysis, while the ray and skull symbols represent the harsh environmental conditions. (**b**) Potential ecological implications of lysogenic to lytic cycle transition. Prophages entering the lytic cycle reprogram the host’s metabolism to favor phage replication, influence the bacterial community structure through phage-mediated host mortality, facilitate horizontal gene transfer, and drive the biogeochemical cycle.

Free phages in the natural environment are under direct stress. For example, Wilhelm et al. found that solar radiation-induced phage decay decreased with increasing seawater depth in the Gulf of Mexico [115]. Lysogeny allows the phage to avoid direct exposure to solar radiation, possibly explaining why lysogeny in offshore areas with high light transmission is more prevalent than in turbid coastal waters [21]. Free phages in the marine environment are also at risk of grazing by heterotrophic nanoflagellates, or sinking by attaching to marine aggregates (Figure 2a) [116,117]. Moreover, the fluctuation of conditions in natural environments may compromise phage lytic infection by inhibiting phage adsorption processes, as depicted in the case of high salinity [43]. The structural constraints for phage adsorption (receptor availability) are usually challenging for successful phage lytic infections [30]. The induced temperate phage may display a distinct production strategy compared to lytic phages. For instance, *Serratia liquefaciens* infected by the temperate phage CP6-1 exhibit a larger phage burst size after a long latent period than when infected by the lytic phage CP6-4 [118]. The phage coexists with the host for a long time during lysogeny, and prophage is considered as a manifestation of a greatly delayed phage latency [21]. An increase in the latent period is an indication of the optimization of the phage reproduction strategy. The increase allows the phage to cope with unfavorable environments and form more viral progeny when conditions improve [119]. Shorter latent periods and smaller burst sizes are prevalent in productive, high-host density environments, while low-density or growth-limited hosts are mostly accompanied by a delayed phage latency [120]. In addition, lysogeny may assist specific phages in intraspecific competition, evidenced in superinfection immunity [95,96]. Collectively, the transition to lysogenic cycles may promote phage survival, reproduction, and competition in unfavorable conditions (Figure 2a).

The transition of a phage’s life cycle from lytic to lysogenic exerts multiple effects on the hosts (Figure 2a). Hosts are directly exempted from immediate phage-mediated mortality and are protected from infections by other homogeneous phages. The predator-prey relationship between phage and host can be viewed as an ongoing arms race. The lysogeny is described as a kind of truce that allows the host and phage to coexist, providing mutual benefits through complex interactions that are distinct from the lytic cycle [1]. The AMGs carried by phages with different life strategies have been proposed to modulate microbial metabolisms in different strategies: “plunder and pillage” and “batten down the hatches” [121]. Specifically, lytic phages use AMGs to hijack host metabolisms and intracellular resources for progeny production [122], while some temperate phages increase bacterial virulence and augment host fitness and resistance to the harsh environment by expressing phage-encoded virulence factors and AMGs [123,124]. Numerous studies postulate that prophages contribute to the pathogenesis of bacteria through the expression of phage-encoded virulence factors [125,126]. The release of virulence factors can occur during lysogeny or after bacterial lysis. For example, Shiga toxins, encoded by temperate phages infecting Shiga toxigenic *E. coli*, are released after the initiation of host lysis [125]. Interestingly, host transcriptional regulators sometimes regulate phage-encoded virulence factors, such as ToxR, ToxT, and TcpP in *V cholerae* that regulate prophage-encoded cholera toxin [127]. Prophages may also carry AMGs that can enhance host metabolic activities and fitness. For instance, the temperate phage SopEΦ increases the production of inducible nitric oxide synthase via the sopE gene it carries, facilitating the production of precursors to the electron receptor nitrate. The precursors in turn boosts the luminal growth of *Salmonella enterica* serotype *Typhimurium* in the inflamed mice intestines through nitrate respiration under anoxic conditions [128]. 

Lysogeny may also endow the host with the ability to respond plastically to environmental changes, thus facilitating the host’s adaptation to extreme environments. For example, prophage FCD38-2 activates the expression of the cell wall protein gene *cwpV* in its host *Clostridium diffificile* [129], thereby facilitating the propagation of phages and promoting bacterial survival through biofilm formation [130]. Wang et al. found that *E. coli* containing prophages CPS-53 and CP4-57 were metabolically more stable than prophage deletion mutants under extreme oxidative, osmotic, or acid-stress conditions [123]. Notably, the proportion of lysogeny increases, carrying significantly more AMGs associated with heavy metal metabolisms in soils with chronic cadmium contamination [85]. Similarly, studies on the extremely high salinity of natural aquatic sites in Senegal and deep-sea hydrothermal vents with stressed physical and chemical parameters implied the prevalence of lysogeny in these extreme environments, and authors inferred that the active AMGs may be one of the important driving factors for the adaptation of bacteria to extreme environments [40,131]. Phage-host seasonal dynamics studies in oligotrophic waters also reveal the prevalence of lysogeny in low primary productivity environments [21,72], suggesting that lysogeny aid host and/or phage survival under nutrient-stressed conditions.

Beside the prophage encoded genes, prophages can also modify the host genome through integration and excision, a phenomenon known as active lysogeny (Figure 2a) [132]. For example, the integration of A118-like prophage leads to the suppression of comK, a gene important for the successful infection of mammalian cells by *Listeria monocytogenes*, thus inhibiting infection. The precise excision of the prophage allows the expression of comK, enabling the bacterium to successfully infect mammalian cells, while the excised prophage re-integrates into the host genome and turns off the comK gene after infection [132,133]. This process benefits both bacteria and phages as it helps bacteria colonize mammalian hosts and improve bacteria and phage survival [132,133]. Imprecise excision of the prophage from the bacterial genome may lead to simultaneous packaging of flanking host sequences inside phage capsids. This packaging is a form of specialized transduction and may help introduce and transfer new phenotypes, such as antibiotic resistance, among bacteria [9]. Different prophages from the same lysogen may share DNA sequence similarities. These regions are targets for homologous recombination and may serve as anchoring points for driving host evolution through prophages-mediated rearrangements of bacterial chromosomes, including inversions and deletions [5,134]. For example, there is a Japanese *Streptococcus pyogenes* M3 strain that differs from an American M3 isolate by two sequential DNA inversions; one inversion was caused by the homologous recombination of two prophages [134]. 

Prophages may be domesticated by the host because of the loss of genes necessary for the production of viral particles and eventually lose the ability to enter the lytic cycle, a phenomenon known as cryptic prophage (Figure 2a) [135]. Of note, the genes of these domesticated prophages can be selectively inherited and maintained by bacteria. The ecological implications of cryptic prophage are gradually being explored [136]. For example, cryptic prophage encoded R-type pyocins with phage-tail-like structures kill other competing bacteria [136,137]. Gene transfer agents (GTA) are bacterial genome-encoded virus-like particles that evolve from mutant prophages that become defective and subsequently decay. GTAs transfer random fragments of the host bacterial genome to recipient cells in a process similar to generalized transduction [138]. Phage-related chromosomal islands (PRCIs) are another class of mobile genetic elements in the bacterial genome that evolved specifically from domesticated prophages [139]. PRCIs are similar to active lysogeny and regulate the bacterial operon through integration and excision processes [140,141]. However, PRCIs differ from active lysogeny because they cannot produce infectious phage particles [132]. The decay of prophages also leads to repetitive sequences that facilitate the insertion of exogenous genes into bacterial chromosomes, forming niche-defining genomic islands [136]. In *Cyanobacteria*, these relic prophages are thought to facilitate the mobility of a gene involved in the nitrogen-stress response [142]. Bacteria may utilize decayed prophages in various ways that exhibit a wide range of ecological significance. The transition from lytic to lysogenic life cycles undoubtedly provides more opportunities for such utilization.

### 3.2. The Transition from Lysogenic to Lytic Cycle

The lytic process is necessary for the majority of phages for phage reproduction and propagation. A single lytic cycle can produce a dozen to hundreds of phage progeny. A mean burst size of 24 and 34 is calculated for phages in marine and freshwater environments, respectively [143]. Phages undergoing the lytic cycle impose a heavy burden on the host to provide resources for the phage burst events, presumably reshaping host metabolisms significantly [122]. Phages employ numerous strategies during the lytic cycle to take over host metabolisms and favor phage replication (Figure 2b). For example, Cristina et al. found that the lytic infection of phage PSA-HS2 in marine bacterium *Pseudoalteromonas* up-regulated the expression of host-encoded nucleotide metabolism gene thyA and chaperone gene groEL/ES to favor phage DNA replication and virion head assembly. In contrast, the lytic infection of phage PSA-HP1 infecting the same host reprogrammed the host’s metabolism to enhance translation and shuttle energy metabolisms by synthesizing sulfur-rich amino acids and degrading them for energy via the glyoxylate-TCA cycle [122]. Similarly, comparative metagenomic analyses carried out by Enav et al. suggested that marine viruses direct host metabolism towards nucleotide biosynthesis upon infection [144]. Hurwitz et al. further postulated that viral infection might trigger a starvation response in the host to drive carbon through non-glycolytic pathways and promote dNTP biosynthesis [145]. The induction of prophages presumably significantly influences the host’s metabolic profile and biochemical composition and hence the composition of lysate, which in turn possibly affect microbial food webs and biogeochemical cycles [146,147].

When prophages enter the lytic cycle, they significantly influence the dynamics of the bacterial community structure (Figure 2b) [6,7]. The KtW model suggests that prophage induction directly affects bacterial abundance and diversity at the community level, where phage predation controls the growth of dominant bacterial populations, thus maintaining the diversity of prokaryote communities [103]. Numerous studies have collectively revealed that prophages induced by various inducers, such as mitomycin C and UV, alter the bacterial community structure [58,59,60,61]. For example, Chen et al. reported a significant increase in the proportion of the early inferior population of *Hyphomicrobiaceae* after mitomycin *C-*mediated induction of prophages in a microcosm experiment using eutrophic coastal waters of Xiamen Bay [111]. Besides, prophage induction under specific circumstances benefits the hosts by enhancing their adaptability in the face of competition. Prophage induction assists the host in killing the competing strains by mediating the release of bacteriocins [130]. For instance, *E. coli* and *S. enterica serovar* Typhimurium produce group B colicins and lack the corresponding export proteins. The cell lysis mediated by prophage induction promotes the release of colicins, inhibiting the competing strains [148]. Moreover, prophage induction in sub-populations within biofilms enhances biofilm formation and maintenance [124,149]. High cell density or abundant reactive oxygen within the biofilm can stimulate prophage induction, enhancing biofilm formation and maintenance through multiple effects, including providing nutrients and extracellular DNA to the adjacent cells and creating hollow centers in the biofilm [150]. Carrolo et al. showed that spontaneous prophage induction promoted DNA release, which in turn enhanced biofilm formation in *Streptococcus pneumoniae* [151]. Rossmann et al. reported that the local lysis of *Enterococcus faecalis* lysogenized cells V583ΔABC enabled the remaining bacteria to benefit from the decreased population density and facilitated the dispersal of bacteria from already established biofilms. In contrast, a mutant deficient in one of the prophages in its genome exhibited a significantly weak dispersal of the biofilm [152]. Prophage induction in individual lysogenic cells is an acceptable cost because the population benefits from it through the reduced cell density and increase in available material and resources [124].

Prophage induction potentially facilitates horizontal gene transfer (HGT) among bacteria and phages (Figure 2b). Phage-mediated HGT is one of the primary driving forces for prokaryote genetic diversity and evolution, with an estimated occurrence of 2 × 10^16^ per second worldwide [5]. Sequence analysis has revealed the occurrence of HGT between cyanobacteria and their phages, probably numerous times, as evidenced by the presence of photosynthesis genes *psbA* and *psbD* in the genome of cyanophages [138]. Phage-induced HGT can occur in diverse forms, either through the release of the host DNA that may be subsequently acquired by surrounding cells [5] or in the form of generalized or specialized transduction [9]. Prophage induction likely provides the active “substrate” for these HGT events to occur with host cell lysis and phage propagation.

Prophage inductions can greatly impact the biogeochemical cycle (Figure 2b). The viral shunt theory proposes that the re-utilization of cell components released by phage-mediated lysis of bacteria promotes the internal recycling of nutrients in the prokaryotic stage, thus reducing energy flow to the higher trophic level [6,153]. It also alters the particulate organic matter (POM) and dissolved organic matter (DOM) content of the environment [7]. Studies estimate that 25% of the carbon fixed through photosynthesis cycles goes through the viral shunt [153]. Most of the released active intracellular components, such as amino acids and nucleic acids, are re-utilized and eventually flow into the carbon dioxide pool by respiration. In contrast, capsular polysaccharides, cell wall components, and membrane-derived proteins such as porins are important and refractory constituents of the DOM pool [30,103]. The inactive carbon components can be exported to deeper oceans, significantly affecting the carbon cycle in the ocean due to the high carbon content ratio of the components [7]. Notably, phage particles formed by components almost entirely derived from bacteria contain higher phosphorus and nitrogen ratios than the host and thus also provide a recycling mechanism for phosphorus and nitrogen [154]. Viral populations potentially constitute a considerable fraction (>5%) of the total dissolved organic phosphorus (DOP) in marine surface waters [154]. Poorvin et al. reported that the phage lytic process is associated with iron cycling in high-nutrient, low-chlorophyll regions of the open ocean. Phage-mediated bacterial lysis can thus regenerate sufficient amounts of dissolved iron to support the growth of the local phytoplankton community [155]. 

## 4. Summary and Prospects

The choice of a phage’s life cycle is crucial in host-phage interactions. The different cycles have different influences on host fate with substantially different ecological implications. In this review, we summarized the effects of nutrients, salinity, aeration, UV, temperature, heavy metals, environmental pollution, superinfection, and host density on phage lysogenic-lytic decisions. The outlined factors affect phage decisions to some extent, but their roles and underlying mechanisms remain largely unknown. The enormous complexity and diversity of regulatory networks of phage life cycle decisions suggest that it is controlled by a “complex combination of factors” rather than a single straight factor. Notably, phage experiments in some studies had no response, while some even had contrasting responses to single factors, suggesting the uncertainty of the sole effects of these factors on the phage life cycle. Nevertheless, the data herein is valuable and provides a framework for elucidating how temperate phages respond to their environments. The data also aids in exploring the dynamics and outcomes of phage-host interactions in continuously changing ecosystems, especially amid the increased anthropogenic activity and global climate change crisis.

The existing studies are limited by several factors from the phage life cycle transition perspective. Firstly, the studies use different approaches, including alteration of the number of phage particles, plaque number, turbidity of the host culture, and assessment of lysogeny using metagenomic techniques to determine the proportion of lysogeny. This difference in methodology makes it difficult to compare the results across different studies [63,68,90]. Secondly, numerous studies only focus on one or few factors, especially in field studies where the geographical and temporal variability involves variations in many environmental factors. The other potential affecting factors remain un- or poorly controlled coupled with a non-extensively evaluation of their influences on the results, thus compromising the reliability of the outcomes [22,153]. It remains unknown whether the response of a cultivated phage-host system to inducers under laboratory conditions reflects the actual situations in natural environments. In the same line, several questions remain unaddressed: does each specific sub-population respond similarly when a factor induces an entire phage community in the environment? How should we analyze and interpret data arising from such environments? Do the situations of a limited set of samples reflect the true picture of a large spatial scale because of the significant heterogeneity in viral abundance and diversity over extremely small spatial scales? [45].

Several studies have explored the molecular mechanisms for lysogeny maintenance and induction in some phage-host models [1,15,156]. Studies on phage λ initially elaborated on the important role of CI and Cro regulators as a bistable genetic switch for lysogenic and lytic states [15]. The molecular mechanisms underlying life cycle transitions were then further explored in phages infecting Gram-positive hosts, which revealed diverse modules and systems involved in lysogeny maintenance and induction. The modules and systems included the λ-like lysogeny modules, the lysogenic system in phages infecting lactic acid bacteria, the protease-encoded lysogeny modules, the lysogeny-regulating arbitrium QS system, and the lysogeny-lysis decision-making system in extrachromosomal temperate tectivirues [1]. In most cases, phages rely directly on the bacterial SOS response for lysogeny induction [1]. However, molecular models have revealed lysogeny inductions not coupled to the host’s SOS response, which rely on phage-encoded receptors to detect host-generated QS autoinducers [113]. The existing knowledge of molecular mechanisms for life cycle transition is still relatively scarce. More paradigms should thus be proposed and merged to enhance an in-depth understanding of the underlying mechanisms of the effects of different factors, given the considerable diversities of phage-host interactions in nature.

The phage life cycle transition has non-negligible implications in phage-host interactions and biogeochemical cycles. Notably, the mechanisms of phage-host interactions are potentially more complex than we envision. The ecological significance of the phage life cycle transition may also go far beyond what this review summarizes because our current knowledge of phages is mainly derived from culturable phage-host systems. Despite the lack of relevant studies, we believe that a greater understanding of how factors regulate phage life cycles could be potentially useful in various fields. These fields include enrichment and purification of specific phages in laboratory settings, control of bacterial contaminations or microbe community in food processing, fermentation, and wastewater treatment processes, and control of phage preparation yield in phage therapy, among other fields.

## Abbreviations

FVIC, frequency of visibly infected cells; UV, ultraviolet radiation; VBR, virus-to-bacteria ratio; PCBs, polychlorinated biphenyls; MOI, multiplicity of infection; KtW, Kill-the-Winner; PtW, Piggyback-the-Winner; QS, quorum sensing; AHLs, acyl-homserine lactones; DPO, 3,5-dimethylpyrazin-2-ol; GTA, Gene transfer agents; PRCIs, Phage-related chromosomal islands; HGT, horizontal gene transfer; POM, particulate organic matter; DOM, dissolved organic matter; DOP, dissolved organic phosphorus.
